# Determinants of household-, maternal- and child-related factors associated with nutritional status among children under five in Mali: evidence from a Demographic and Health Survey, 2018

**DOI:** 10.1017/S1368980024000363

**Published:** 2024-02-05

**Authors:** Tafere Gebreegziabher, Saran Sidibe

**Affiliations:** Food Science and Nutrition, Department of Health Sciences, Central Washington University, 400 E University Way, Ellensburg, WA 98926, USA

**Keywords:** Undernutrition, Stunting, Wasting, Underweight, Risk factors

## Abstract

**Objective:**

The current study aims to determine household-, maternal- and child-related factors influencing nutritional status among children under five in Mali.

**Design:**

Quantitative cross-sectional study using secondary data extracted from Mali DHS-VI 2018.

**Setting:**

Urban and rural areas of Mali.

**Participants:**

A total of 8908 children participated, with 3999 in the younger age group (0–24 months) and 4909 in the older age group (25–59 months).

**Results:**

In the younger age group, the prevalence of stunting, wasting and underweight was 18·8 % (95 % CI%: 17·5, 20·0), 24·6 % (95 % CI: 23·2, 26·0) and 13·2 % (95 % CI: 12·1, 14·3), respectively, while in the older age group, it was 24·9 % (95 % CI: 23·7, 26·2), 22·7 % (95 % CI: 21·5, 24·0) and 5·7 % (95 % CI: 5·0, 6·5), respectively. Being average or large size at birth, having piped source of water, receiving Zn, deworming, high maternal BMI, receiving Fe during pregnancy, higher maternal education and being rich were associated with lower odds of one or more form of undernutrition in both groups. On the other hand, children who were anaemic, drank from a bottle, maternal anaemia, current pregnancy of mothers and living in rural areas were associated with higher odds of stunting, wasting or underweight. Interestingly, children who received Fe supplementation had a higher odds of wasting in the younger group but lower odds of all forms of undernutrition in the older group.

**Conclusions:**

This study emphasised the potential risk factors associated with undernutrition in children. Children who consume non-potable water, have mothers with lower levels of education and BMI and reside in rural areas are more likely to experience undernutrition.

Childhood undernutrition persists as a significant public health concern, especially in low-income countries. Despite ongoing initiatives, such as micronutrient supplementation, supplementary feeding programs and the promotion of hygiene and sanitary food practices the challenge endures, with sub-Saharan Africa (SSA) experiencing a particularly pronounced impact^(1,2)^. Globally, the prevalence of stunting has decreased from 33 % in 2000 to 22·3 % in 2022, and in SSA, it has reduced from 34·5 % to 31·1 %. However, despite this encouraging decline on a global scale, progress in SSA has fallen short of meeting the global target. In fact, SSA is the only sub-region where the absolute number of children affected by stunting has increased in recent years^(3)^. Not only stunting but also wasting and underweight, the other forms of undernutrition, have exhibited similar trends. According to Akombi et al., the average prevalence of wasting and underweight among children under five in SSA was 12 % and 24·8 %, respectively. Specifically in Mali, the rates of stunting, wasting and underweight were reported as 38·3 %, 12·7 % and 25 %, respectively^(1)^.

Undernutrition has a negative impact on the efficiency of countries and gives rise to economic and societal difficulties within vulnerable populations. It is linked to substandard brain growth, leading to detrimental effects on cognitive development, academic achievements and economic output during adulthood^(4)^. Children under five are particularly susceptible to the consequences of undernutrition, experiencing significant morbidity and mortality as a result of a weakened immune system. It is estimated that undernutrition is the underlying cause of child mortality in about 45 % of all deaths reported for children under 5 years of age^(5)^. The link between malnutrition and infections may be influenced, to some extent, by the confounding effect of poverty, which serves as a common determinant. However, there is also a potential two-way causal relationship: malnutrition heightens susceptibility to infections, while infections exacerbate malnutrition by diminishing appetite, promoting catabolism and intensifying the body’s demand for nutrients^(6)^.

Over the years, various contributing factors to undernutrition have been reported. These factors include, but not limited to, socio-economic and demographic factors, physical factors, nutritional factors, hygiene and sanitation factors, medical factors, as well as environmental factors^(1,7–9)^. A study conducted in Mali has indicated a significant correlation between wasting and clinical malaria^(10)^. Mali is one of the countries situated in the African meningitis belt, and meningitis has been associated with undernutrition due to reduced immune system^(11,12)^. In the agricultural regions of Mali, diarrhoea, preterm birth and a low dietary diversity score have been identified as factors associated with chronic malnutrition in children aged 6–24 months^(13)^. Additionally, another study conducted in rural Mali demonstrated that increased access to toilet facilities has led to improvements in growth, particularly among children under 2 years old^(14)^.

Undernutrition is a result of many factors that affect child growth and development. It is important to explore all possible ways to understand the nature and complex interplay between undernutrition and the contributing factors in order to find a sustainable solution. While there may be similarities, problems can vary from region to region, even between rural and urban areas within the same country. In Mali, for instance, food variety and diet diversity were found to be associated with the nutritional status of children aged 6–59 months in urban areas. However, these two factors did not show any association among children living in rural areas^(15)^.

In our previous study in Ethiopia, we examined the associated factors of undernutrition in children separately, considering two distinct age groups: 0–24 months and 25–59 months^(7)^. However, to the best of our knowledge, no nationwide study has been conducted to date that specifically analyses undernutrition in these two age groups of children in Mali. Therefore, the primary objective of the current study is to investigate the varying impacts of three categories of predictors (household, maternal and child variables) on the nutritional status of children in the younger age group (0–24 months) and the older age group (25–59 months). By addressing this research gap, we aim to contribute to a better understanding of the factors influencing undernutrition in different age groups, which can inform targeted interventions and policies to improve the nutritional outcomes of children in Mali.

## Materials and methods

### Study setting

We conducted a secondary data analysis based on the Mali Demographic and Health Survey (DHS) for 2018. The 2018 survey is a national survey designed to provide data for monitoring the population and health situation in Mali. It is the sixth Demographic and Health Survey conducted in Mali since 1987 and was implemented by *l’Institut National de la Statistique* (INSTAT) in close collaboration with the *Cellule de Planification et de Statistique Secteur Santé-Développement Social et Promotion de la Famille* (CPS/SS-DS-PF). The DHS Program, an initiative backed by USAID, received assistance from ICF in the form of technical support. This support aimed to facilitate population and health surveys in various countries across the globe. A total of 8908 cases based on the children’s age were used for the current analysis, among which 3999 children were aged 0–24 months, and 4909 children were aged 25–59 months. All children under age 5 years of age who were eligible for height and weight measurements were eligible for the DHS data collection.

The DHS Program has established standardised procedures, methodologies and manuals to oversee the survey process. Rigorous steps are taken to ensure accurate representation and comparability of data across countries. Data collection involves questionnaires, manuals, biomarkers and geographic information systems. The sampling strategy includes national, urban–rural and regional representation, employing a stratified two-stage cluster design. Enumeration areas are initially selected from Census files, followed by the second stage where households are sampled from an updated list within each selected enumeration area.

### Variables and measures

Dependent variables: The dependent variables were stunting, wasting and underweight. The children were considered stunted, wasted or underweight if the height-for-age Z-score, the weight-for-age Z-score or the weight-for-height Z-score was less than −2 sd using the new WHO child growth standards^(16)^.

Independent variables: We employed a total of twenty two factors to explain stunting, wasting and underweight. These factors were categorised into three main groups: child characteristics (consisting of nine variables), maternal factors (comprising seven variables) and socio-demographic variables (comprised of six variables). The independent variables were selected based on review of literature and the UNICEF conceptual frame work^(17)^. The household and socio-economic characteristics included in the analyses were explained as follows. Household index served as a substitute for household income and was evaluated using an asset-based index. This index integrated data regarding ownership of consumer goods, housing conditions, as well as water and sanitation amenities to estimate the wealth level of the household^(18)^. Wealth index was categorised as poorest, poorer, middle, richer and richest. Other factors included were area of residence, total number of children ever born, the employment status of the mothers, maternal education and household head. Among the maternal factors considered were the current age and height of mothers, whether the mother received Fe supplements during pregnancy, her current pregnancy status, breastfeeding status, maternal anaemia level and BMI. BMI was computed using height and weight and was defined as BMI value below 18·5 kg/m^2^ (underweight), 18·5–24·9 kg/m^2^ (normal weight), 25–29·9 kg/m^2^ (overweight) and ≥ 30·0 kg/m^2^ (obese). The child factors were sex of child, size at birth, anaemia level, source of drinking water, if child drank from bottle the night preceding the survey, if child was taking Fe pills, sprinkles or syrup, drugs for intestinal parasite in the last 6 months, if child has diarrhoea recently and if child was given Zn for diarrhoea treatment.

### Statistical analysis

We conducted data analysis using IBM SPSS Statistics version 23, a statistical software package. All analyses were weighted to account for sampling probabilities. Descriptive analysis was performed to examine the characteristics of the study sample. To investigate the relationships between the aforementioned explanatory variables and the three outcome variables, multivariable binary logistic regression analyses were employed. For each of the two age groups (0–24 months and 25–59 months), three separate regression models were constructed. The selection of explanatory variables for the regression analysis was performed based on previous research findings and the UNICEF conceptual framework^(17)^. To assess multicollinearity among the explanatory variables, we utilised the variance inflation factor, and variables with a variance inflation factor exceeding 10 were eliminated from the analysis. OR and 95 % CI were calculated to determine the likelihood of being undernourished associated with each factor in the logistic regression model. Statistical significance was considered if the *P* value was < 0·05.

## Results

### Characteristics of study participants

**Fig. 1:**
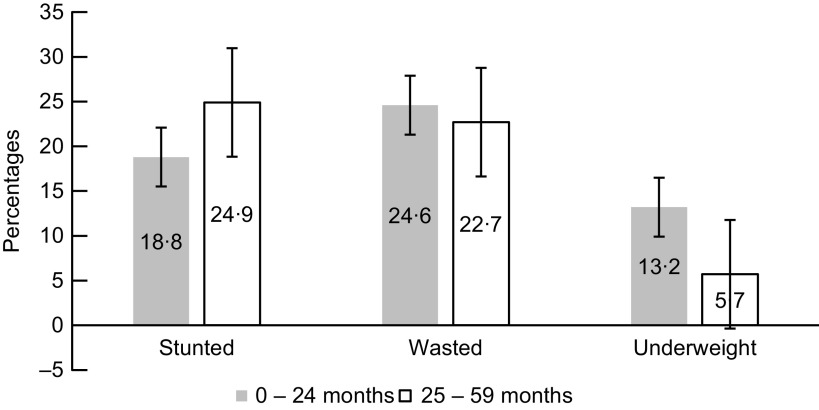
Prevalence of stunting, wasting and underweight of children, with their 95 % confidence intervals represented by vertical bars

As shown in Table 1, the majority of the children in the age group 0–24 months and of the children in the age group 25–59 months were females. Nearly 17 % of the younger age group and 13·3 % of the older age group were small size at birth. Anaemia was highly prevalent in both age groups with almost 88 % in the younger and 76 % in the older age groups. Most of the children were moderately anaemic in both age groups. Of the children, 15·1 % in the younger and 14·7 % in the older age group received Zn supplement for diarrhoea treatment, 18·8 % in the younger and 22·6 % in the older age group received Fe supplement, and nearly 55 % in the younger and 66 % in the older age group received vitamin A supplement in the last 6 months preceding the survey. Roughly the same percentage (67 %) of children in both age groups lack access to a piped water source. In the younger age group, 35·8 % experienced sickness such as fever, cough and/or shortness of breath in the last 6 months, while in the older age group, the percentage was 27·3 %. Most of the children in both age groups have had vaccination, with 56·7 % in the younger age group and 71·3 % in the older age group. In comparison to the younger age group, a significant percentage of the older age group—27·2 % and 42·0 %, respectively—had received medication for intestinal parasites in the last 6 months preceding data collection. While having diarrhoea recently was high in the younger age group. Prevalence of stunting, wasting and underweight in both groups was 22·2 % (95 % CI: 21·2, 23·1), 23·6 % (95 % CI: 22·7, 24·5) and 9·1 % (95 % CI: 8·4, 9·7), respectively (Fig. 1).

**Table 1:** Nutrition, health and demographic characteristics of children aged 0–24 months and 25–59 months. Demographic and health survey data, Mali, 2018 (*n* 8908)

	0–24 months (*n* 3999)		25–59 months (*n* 4909)		Total	
Variable	*n*	%	*n*	%	*n*	%
Child sex						
Male	2050	51·3	2490	50·7	4540	51
Female	1949	48·7	2419	49·3	4368	49
Anaemia*						
Severe (Hb < 7 g/dl)	92	6·4	107	4·4	199	5·1
Moderate (Hb = 7–9–9 g/dl)	880	61·5	1080	44·2	1960	50·6
Mild (Hb = 10–10·9 g/dl)	286	20·0	664	27·2	950	24·5
Not anaemic (Hb ≥ 11 g/dl)	173	12·1	592	24·2	765	19·8
Size of child at birth						
Small	633	16·7	619	13·3	1252	14·8
Average	1903	50·1	2405	51·6	4308	51·0
Large	1262	33·2	1633	35·1	2895	34·2
Given Zn tablets for diarrhoea treatment†						
No	770	84·9	546	85·3	1316	85·1
Yes	137	15·1	94	14·7	231	14·9
Child morbidity‡						
No	2560	64·2	3554	72·7	6114	68·9
Yes	1429	35·8	1337	27·3	2766	31·1
Vitamin A supplement in the last 6 months						
No	1795	45·2	1668	34·2	3463	39·2
Yes	2174	54·8	3203	65·8	5377	60·8
Fe supplement§						
No	3192	81·2	3720	77·4	6912	79·1
Yes	741	18·8	1089	22·6	1830	20·9
Deworming||						
No	2829	72·3	2765	58·0	5594	64·4
Yes	1086	27·7	2005	42·0	3091	35·6
Ever had vaccination						
No	713	43·3	251	28·7	964	38·2
Yes	935	56·7	624	71·3	1559	61·8
Source of drinking water						
Not piped	2662	66·6	3290	67·0	5952	66·8
Piped	1337	33·4	1619	33·0	2953	33·2
Had diarrhoea recently						
No	3079	77·1	4241	86·7	7320	82·4
Yes	914	22·9	651	13·3	1565	17·6

* Included children aged 6–59 months.

† Zn tablet given any time since started diarrhoea.

‡ Child had one or more of the listed health problems in the last 2 weeks (fever, cough and short rapid breath).

§ Taking Fe pills, sprinkles or syrup.

|| Drugs for intestinal parasite in last 6 months.

Table 2 shows that the majority of the mothers were in the age category between 25 and 34 years and had a normal BMI. Among the mothers, the majority were rural inhabitants and had no education. The prevalence of anaemia was high in both groups, with 62·3 % in the younger age group and 64·5 % in the older age group. More than 39 % of the younger age group and over 41 % of the older age group were classified as ‘poor’, while a similar proportion of women were categorised as ‘rich’ based on the wealth index. Of the mothers, nearly 70 % in the younger age group and 74 % in the older age group had received Fe supplements during pregnancy.

**Table 2:** Health, socio-economic and demographic characteristics of mothers by age group of children. Demographic and Health Survey data, Mali, 2018 (*n* 8908)

	0–24 months (*n* 3999)		25–59 months (*n* 4909)	
Variable	*n*	%	*n*	%
Mother’s age				
15–14	1432	35·8	1177	24·0
25–34	1817	45·4	2503	51·0
35–49	750	18·8	1229	25·0
Mother’s BMI (kg/m^2^)				
< 18·5	181	9·0	160	6·3
18·5–24·9	1276	63·3	1527	60·7
25–29·9	373	18·2	550	21·9
≥ 30·0	187	9·3	279	11·1
Short stature (< 150 cm)				
No	49	2·4	51	2·0
Yes	1968	97·6	2465	98·0
Total children ever born				
1–3	2015	50·4	2031	41·4
4–6	1327	33·2	1922	39·1
7–15	656	16·4	956	19·5
Fe given during pregnancy				
No	138	30·2	535	26·0
Yes	319	69·8	1525	74·0
Anaemia				
Severe (Hb < 8 g/dl)	72	3·7	93	3·8
Moderate (Hb = 8–10·9 g/dl)	665	33·8	866	35·1
Mild (Hb = 11–11·9 g/dl)	493	25·0	632	25·6
Not anaemic (≥ 12 g/dl)	739	37·5	876	35·5
Mother’s education				
No education	2820	70·5	3667	74·7
Primary education	507	12·7	587	12·0
Secondary	614	15·3	599	12·2
Higher	58	1·5	56	1·1
Place of residence				
Urban	1029	25·7	1274	26·0
Rural	2970	74·3	3635	74·0
Wealth index				
Poorest	740	18·5	1033	21·0
Poorer	834	20·9	985	20·1
Middle	861	21·5	1042	21·2
Richer	822	20·6	999	20·4
Richest	742	18·6	850	17·3

### Correlates of childhood undernutrition in Mali

#### Child factors

As shown in Table 3, birth size, anaemia, source of drinking water, drinking from a bottle, receiving Zn supplement, receiving Fe supplement and receiving drugs for intestinal parasite were associated with one or more of the three forms of childhood undernutrition. In both age groups, the likelihood of stunting, wasting or being underweight was lower in children with average or large size at birth. In the younger age group, anaemic children were more likely to be wasted (OR = 2·5; 95 % CI 1·23, 4·95), while in the older age group, they were more likely to be stunted (OR = 2·02; 95 % CI 1·57, 2·60). Children living in households with a piped source of drinking water were less likely to experience stunting (OR = 0·63; 95 % CI 0·52, 0·76), wasting (OR = 0·69; 95 % CI 0·58, 0·81) or being underweight (OR = 0·76; 95 % CI 0·62, 0·93) in the younger age group. They were also less likely to experience stunting (OR = 0·51; 95 % CI 0·44, 0·60) or wasting (OR = 0·59; 95 % CI 0·50, 0·69) in the older age group.

**Table 3:** Factors associated with undernutrition in children aged 0–24 months and 25–59 months: child factors. Demographic and Health Survey data, Mali, 2018 (*n* 8908)

	0–24 months (*n* 3999)						25–59 months (*n* 4909)					
	Stunting		Wasting		Underweight		Stunting		Wasting		Underweight	
	OR	95 % CI	OR	95 % CI	OR	95 % CI	OR	95 % CI	OR	95 % CI	OR	95 % CI
Size at birth												
Small	1·00	Ref	1·00	Ref	1·00	Ref	1·00	Ref	1·00	Ref	1·00	Ref
Average	0·72***	0·58, 0·90	0·68***	0·55, 0·83	0·58***	0·45, 0·75	0·99	0·80, 1·22	0·75**	0·64, 0·93	0·60**	0·44, 0·82
Large	0·59***	0·46, 0·76	0·52***	0·42, 0·66	0·53***	0·41, 0·70	0·81	0·65, 1·00	0·54***	0·43, 0·67	0·38***	0·27, 0·55
Sex												
Male	1·00	Ref	1·00	Ref	1·00	Ref	1·00	Ref	1·00	Ref	1·00	Ref
Female	1·19	0·70, 2·04	1·28	0·73, 2·26	1·35	0·69, 2·62	1·60	0·93, 2·75	1·61	0·87, 2·95	2·45	0·70, 8·54
Anaemia†												
Not anaemic	1·00	Ref	1·00	Ref	1·00	Ref	1·00	Ref	1·00	Ref	1·00	Ref
Anaemic	1·12	0·76, 1·66	2·5*	1·23, 4·95	0·84	0·55, 1·29	2·02***	1·57, 2·60	2·33	1·77, 3·05	1·02	0·67, 1·53
Source of water												
Not piped	1·00	Ref	1·00	Ref	1·00	Ref	1·00	Ref	1·00	Ref	1·00	Ref
Piped	0·63***	0·52, 0·76	0·69***	0·58, 0·81	0·76***	0·62, 0·93	0·51***	0·44, 0·60	0·59***	0·50, 0·69	0·79	0·61, 1·04
Drank from bottle												
No	1·00	Ref	1·00	Ref	1·00	Ref	1·00	Ref	1·00	Ref	1·00	Ref
Yes	0·82	0·59, 1·14	1·10	0·84, 1·45	0·80	0·55, 1·17	0·59	0·24, 0·83	1·92*	1·03, 3·40	5·31**	1·55, 18·24
Given Zn‡												
No	1·00	Ref	1·00	Ref	1·00	Ref	1·00	Ref	1·00	Ref	1·00	Ref
Yes	0·43*	0·18, 0·50	0·56*	0·35, 0·91	0·79	0·47, 1·31	0·62	0·28, 1·39	0·38*	0·17, 0·83	0·91	0·40, 2·09
Given Fe§												
No	1·00	Ref	1·00	Ref	1·00	Ref	1·00	Ref	1·00	Ref	1·00	Ref
Yes	1·17	0·95, 1·44	1·20*	1·00, 1·35	1·08	0·85, 1·34	0·83*	0·70, 0·99	0·24**	0·11, 0·53	0·72***	0·62, 0·84
Deworming||												
No	1·00	Ref	1·00	Ref	1·00	Ref	1·00	Ref	1·00	Ref	1·00	Ref
Yes	1·45	0·76, 2·75	0·75	0·56, 1·01	0·81	0·51, 1·26	0·87	0·46, 1·63	0·62**	0·45, 0·86	0·27***	0·14, 0·53
Diarrhoea¶												
No	1·00	Ref	1·00	Ref	1·00	Ref	1·00	Ref	1·00	Ref	1·00	Ref
Yes	1·44***	1·19, 1·73	1·77***	1·5, 2·1	1·73***	1·41, 2·13	1·59***	1·3, 1·91	1·9***	1·56, 2·26	1·55**	1·14, 2·12

Ref., reference category.

* *P* < 0·05.

** *P* < 0·01.

*** *P* < 0·001.

† Not anaemic, Hb ≥ 11 g/dl; anaemic, Hb < 11 g/dl.

‡ Zn given for diarrhoea treatment.

§ Taking Fe pills, sprinkles or syrup.

|| Drugs for intestinal parasite in last 6 months.

¶ Had diarrhoea recently.

The children in the older age group who drank from a bottle had higher odds of experiencing wasting (OR = 1·92; 95 % CI 1·03, 3·40) or being underweight (OR = 5·31; 95 % CI 1·55, 18·24). Furthermore, children who had diarrhoea recently were more likely to experience stunting, wasting and underweight in both age groups. The odds of stunting or wasting were lower by 57 % and 44 %, respectively, in the younger age group who received Zn supplements for diarrhoea treatment. In the older age group who received Zn supplements for diarrhoea treatment, the odds of wasting were lower by 62 %. Children in the younger age group who received Fe supplement were 1·20 times more likely to experience wasting (95 % CI 1·00, 1·35). Conversely, in the older age group, children were less likely to experience stunting (OR = 0·83; 95 % CI 0·70, 0·99), wasting (OR = 0·24; 95 % CI 0·11, 0·53) or being underweight (OR = 0·72; 95 % CI 0·62, 0·84). Furthermore, children in the older age group who received medication for intestinal parasite were less likely to experience wasting (OR = 0·62; 95 % CI 0·45, 0·86) or be underweight (OR = 0·27; 95 % CI 0·14, 0·53).

#### Maternal health, nutrition and physiological factors

According to the findings presented in Table 4, several factors demonstrated associations with at least one of the three types of childhood undernutrition. For the younger age group, children whose mothers fell into the overweight or obese category for BMI had lower odds of experiencing stunting, wasting or underweight. Similarly, among children in the younger age group, those whose mothers had a normal BMI had lower odds of experiencing wasting or underweight. Likewise, in the older age groups, children whose mothers had a normal BMI had lower odds of experiencing stunting or underweight. In contrast, among the older age groups, children whose mothers had short stature were 1·24 times more likely to experience stunting (95 % CI 1·06, 1·44), while children whose mothers were anaemic were 1·47 times more likely to experience stunting (95 % CI 1·04, 2·08).

**Table 4:** Factors associated with undernutrition in children aged 0–24 months and 25–59 months: maternal health and nutrition factors. Demographic and Health Survey, Mali, 2018 (*n* 8908)

	0–24 months (*n* 3999)						25–59 months (*n* 4909)					
	Stunting		Wasting		Underweight		Stunting		Wasting		Underweight	
	OR	95 % CI	OR	95 % CI	OR	95 % CI	OR	95 % CI	OR	95 % CI	OR	95 % CI
Age (years)												
15–24	1·00	Ref	1·00	Ref	1·00	Ref	1·00	Ref	1·00	Ref	1·00	Ref
25–29	0·95	0·72, 1·25	1·03	0·80, 1·33	0·97	0·71, 1·33	0·98	0·64, 1·50	1·45	0·94, 2·22	1·41	0·69, 2·05
≥ 30	1·01	0·70, 1·46	1·02	0·74, 1·43	1·04	0·69, 1·57	1·10	0·69, 1·73	1·31	0·82, 2·07	0·94	0·43, 2·05
BMI (kg/m^2^)												
< 18·5	1·00	Ref	1·00	Ref	1·00	Ref	1·00	Ref	1·00	Ref	1·00	Ref
18·5–24·9	0·72	0·49, 1·05	0·56***	0·39, 0·79	0·43***	0·29, 0·64	0·47*	0·26, 0·86	0·64	0·35, 1·16	0·39*	0·17, 0·90
25–29·9	0·42***	0·25, 0·69	0·47***	0·31, 0·72	0·56*	0·35, 0·91	0·37**	0·19, 0·72	0·32	0·16, 0·63	0·51	0·20, 1·28
≥ 30	0·50*	0·27, 0·94	0·49**	0·28, 0·83	0·39**	0·20, 0·75	0·19***	0·08, 0·45	0·29	0·13, 0·66	0·59	0·21, 1·65
Short stature (< 150 cm)												
No	1·00	Ref	1·00	Ref	1·00	Ref	1·00	Ref	1·00	Ref	1·00	Ref
Yes	0·79	0·37, 1·70	0·65	0·33, 1·29	0·64	0·29, 1·42	1·24**	1·06, 1·44	1·36	0·86, 2·15	0·44	0·12, 1·65
Anaemia†												
Not anaemic	1·00	Ref	1·00	Ref	1·00	Ref	1·00	Ref	1·00	Ref	1·00	Ref
Anaemic	0·99	0·76, 1·29	0·94	0·74, 1·18	1·29	0·96, 1·75	1·47*	1·04, 2·08	1·19	0·85, 1·66	1·22	0·72, 2·09
Currently pregnant												
No	1·00	Ref	1·00	Ref	1·00	Ref	1·00	Ref	1·00	Ref	1·00	Ref
Yes	2·04**	1·28, 3·26	2·04**	1·28, 3·26	0·85	0·38, 1·87	1·21	0·84, 1·75	1·06	0·74, 1·53	0·52	0·25, 1·07
Fe given during pregnancy												
No	1·00	Ref	1·00	Ref	1·00	Ref	1·00	Ref	1·00	Ref	1·00	Ref
Yes	0·96	0·72, 1·28	0·77*	0·59, 0·99	0·71*	0·52, 0·96	0·58**	0·42, 0·82	0·60**	0·43, 0·84	0·56*	0·34, 0·96
Currently breast-feeding												
No	1·00	Ref	1·00	Ref	1·00	Ref	1·00	Ref	1·00	Ref	1·00	Ref
Yes	0·74	0·57, 1·02	0·29*	0·10, 0·82	0·88	0·54, 1·45	0·92	0·67, 1·26	1·01	0·81, 1·23	1·57	0·77, 3·19

Ref., reference category.

* *P* < 0·05.

** *P* < 0·01.

*** *P* < 0·001.

† Not anaemic, Hb ≥ 11 g/dl; anaemic, Hb < 11 g/dl.

Among the younger age group, children whose mothers were pregnant were 2·04 times more likely to experience stunting and wasting (95 % CI 1·28, 3·26). However, the odds of wasting were lower by 61 % in the younger age group whose mothers were currently breast-feeding (95 % CI 0·10, 0·82). Receiving Fe supplements during pregnancy was another significant predictor of undernutrition, where the likelihood of wasting decreased by 23 % (OR = 0·77; 95 % CI 0·59, 0·99), and the likelihood of being underweight decreased by 29 % (OR = 0·71; 95 % CI 0·52, 0·96) in the younger age group. Furthermore, in the older age group, the likelihood of experiencing stunting, wasting or being underweight decreased by 42 % (OR = 0·58; 95 % CI 0·42, 0·82), 40 % (OR = 0·60; 95 % CI 0·43, 0·84) and 44 % (OR = 0·56; 95 % CI 0·34, 0·96), respectively.

#### Household socio-economic and demographic factors

The results presented in Table 5 demonstrate significant associations between various factors and the occurrence of childhood undernutrition across three different forms. Maternal education, household wealth index, area of residence, household size and mother’s work all displayed statistically significant relationships with at least one form of childhood undernutrition.

**Table 5:** Factors associated with undernutrition in children aged 0–24 months and 25–59 months: household socio-economic and demographic factors. Demographic and Health Survey, Mali, 2018 (*n* 8908)

	0–24 months (*n* 3999)						25–59 months (*n* 4909)					
	Stunting		Wasting		Underweight		Stunting		Wasting		Underweight	
	OR	95 % CI	OR	95 % CI	OR	95 % CI	OR	95 % CI	OR	95 % CI	OR	95 % CI
Maternal education												
No	1·00	Ref	1·00	Ref	1·00	Ref	1·00	Ref	1·00	Ref	1·00	Ref
Primary	0·76	0·57, 1·01	0·83	0·64, 1·07	0·97	0·71, 1·32	0·99	0·79, 1·24	1·03	0·82, 1·29	0·89	0·58, 1·37
Secondary	0·64**	0·46, 0·89	0·68*	0·51, 0·91	1·30	0·74, 1·45	0·55***	0·40, 0·75	0·54***	0·39, 0·73	0·86	0·55, 1·35
Higher	0·49	0·16, 1·45	0·24*	0·07, 0·79	0·63	0·21, 1·87	0·22*	0·05, 0·94	0·38	0·13, 1·13	0·57*	0·33, 0·91
Wealth index												
Poor	1·00	Ref	1·00	Ref	1·00	Ref	1·00	Ref	1·00	Ref	1·00	Ref
Middle	1·02	0·83, 1·27	0·81*	0·66, 0·98	0·93	0·72, 1·19	0·75***	0·61, 0·87	0·78**	0·65, 0·94	0·75	0·53, 1·06
Rich	0·6***	0·45, 0·89	0·69**	0·54, 0·98	0·79	0·58, 1·07	0·55***	0·45, 0·68	0·65***	0·53, 0·81	0·66	0·45, 0·97
Area of residence												
Urban	1·00	Ref	1·00	Ref	1·00	Ref	1·00	Ref	1·00	Ref	1·00	Ref
Rural	0·9	0·67, 1·22	1·24**	0·95, 1·41	0·98	0·72, 1·34	1·21**	0·98, 1·40	1·18	0·93, 1·49	0·61**	0·42, 0·88
Parity												
1–3	1·00	Ref	1·00	Ref	1·00	Ref	1·00	Ref	1·00	Ref	1·00	Ref
4–6	0·92	0·77, 1·11	0·97	0·82, 1·15	0·88	0·71, 1·09	0·86	0·74, 0·11	0·78**	0·67, 0·91	0·65**	0·49, 0·85
≥ 7	0·79	0·53, 1·19	1·07	0·75, 1·52	1·08	0·71, 1·66	0·97	0·73, 1·31	0·81	0·59, 1·11	0·77	0·45, 1·34
Mother’s working												
No	1·00	Ref	1·00	Ref	1·00	Ref	1·00	Ref	1·00	Ref	1·00	Ref
Yes	0·95	0·80, 1·13	0·89	0·77, 1·05	0·91	0·82, 1·21	0·86*	0·75, 0·99	0·95	0·82, 1·09	0·90	0·77, 1·28
Household head												
Male	1·00	Ref	1·00	Ref	1·00	Ref	1·00	Ref	1·00	Ref	1·00	Ref
Female	1·14	0·89, 1·47	1·22	0·97, 1·53	0·99	0·74, 1·33	1·07	0·87, 1·33	0·95	0·82, 1·09	0·84	0·55, 1·28

Ref., reference category.

* *P* < 0·05.

** *P* < 0·01.

*** *P* < 0·001.

In the regression model for the younger age group, children whose mothers had attained secondary or higher education exhibited lower odds of experiencing stunting or wasting compared with those whose mothers had no education. Specifically, the likelihood of stunting or wasting was found to be 36 % lower (OR = 0·64; 95 % CI 0·46, 0·89) and 32 % lower (OR = 0·68; 95 % CI 0·51, 0·91), respectively, for children whose mothers had secondary education. Additionally, the odds of wasting were lower by 76 % (OR = 0·24; CI 0·07, 0·79) for children whose mothers had higher education. Similarly, in comparison with children with economically disadvantaged mothers, children from wealthier households demonstrated a reduced likelihood of experiencing stunting or wasting. Specifically, the odds of stunting were 40 % lower (OR = 0·6; 95 % CI 0·45, 0·89), and the odds of wasting were 31 % lower (OR = 0·69; 95 % CI 0·54, 0·98) for children from wealthier households.

These associations were also observed in the older age group. Furthermore, the analysis revealed that the area of residence was a significant predictor of childhood undernutrition. Children residing in rural areas exhibited 1·24 times higher odds (95 % CI 0·95, 1·41) of experiencing stunting compared with children living in urban areas within the younger age group and 1·21 times higher odds (95 % CI 0·98, 1·40) within the older age group. Among the older age group, children from households where the number of children ever born was between four and six had a 22 % (OR = 0·78; 95 % CI 0·67, 0·91) lower odds of being wasted and a 35 % (OR = 0·65; 95 % CI 0·49, 0·85) lower odds of being underweight. Additionally, the odds of experiencing stunting were 14 % lower in older children whose mothers were employed (OR = 0·86; 95 % CI 0·75, 0·99).

## Discussion

Using nationally representative secondary data, we present the prevalence of the three forms of undernutrition and predictors for each dependent variable, stratifying the children into two age groups. The prevalence of stunting (chronic undernutrition) and underweight has shown a promising decline among children under 5 years old. However, the prevalence of wasting (acute undernutrition) remains high compared with previous studies. A previous study utilising DHS data from 2006 to 2016 reported a prevalence of 38·3 % for stunting, 12·7 % for wasting and 25 % for underweight in children under 5 years old in Mali^(1)^. In our current study, the respective prevalence was 22 %, 24 % and 9·5 %. Although progress has been made in reducing the prevalence of stunting, further efforts are needed to meet the global sustainable development goal of reducing chronic undernutrition by 40 % and acute undernutrition to less than 5 % by the year 2025^(19)^. Childhood undernutrition, particularly stunting, has gained significant attention recently for two reasons. First, a large number of children worldwide are affected by this issue. Second, ample evidence confirms that stunting is associated with poor cognition, educational underperformance and diminished economic productivity later in life. Hence, addressing childhood undernutrition, especially stunting, is crucial not only for the immediate health and well-being of children but also for their long-term development and future opportunities^(20)^.

Childhood undernutrition is a complex issue influenced by various factors, and its consequences are severe and multifaceted, particularly among children under the age of five. In this analysis, we have presented a comprehensive assessment of the potential contributing factors to childhood undernutrition, categorising them into child health and nutrition factors, maternal health and nutrition factors, as well as household socio-economic and demographic factors. Unlike in previous studies^(7,8)^, the gender of the child did not show a significant association with any of the three undernutrition indicators in either age group.

However, child weight at birth was consistently associated with all forms of undernutrition in both age groups. In the younger age group, children with average or large birth weights had lower odds of experiencing stunting, wasting or underweight compared to those with small birth weights. A similar pattern was observed in the older age group, except that stunting was not significantly associated. The association between birth weight and undernutrition has been consistently observed in various regions worldwide^(7,21–23)^. Low birth weight has been identified as a major risk factor for child morbidity and mortality^(24)^. To achieve the Sustainable Development Goal 3·2 target of ending preventable deaths of children under the age of five by 2030, it is crucial to aggressively intervene in enhancing birth weight through improvements in maternal and child nutrition^(25)^.

Anaemia is highly prevalent in Mali, with approximately 82 % of children (aged 6–59 months) and 64 % of mothers (aged 15–49 years) affected, as indicated in this study. Furthermore, anaemia is significantly associated with undernutrition. Among anaemic children, the odds of experiencing wasting were 2·5 times higher in the younger age group, while the odds of experiencing stunting were 2·02 times higher in the older age group. Evidence shows that anaemia diminishes the transportation of oxygen within the body, potentially leading to long-lasting implications for the growth and development of young children^(26,27)^. Additionally, children between the ages of 25 and 29 months born to anaemic mothers had a 1·47 times higher likelihood of experiencing stunting. Maternal anaemia, particularly during pregnancy, has been associated with an increased risk of infant stunting, low birth weight and childhood anaemia^(28,29)^. The causes of anaemia are multifactorial, including micronutrient deficiencies^(30)^, acute and chronic inflammation^(31)^ and disorders affecting Hb synthesis and erythrocyte production^(32)^. However, approximately 50 % of anaemia cases are due to Fe deficiency^(33,34)^. Fe plays a vital role in essential bodily functions, ranging from basic processes like breathing and immune response to complex cellular activities such as DNA creation and cell growth promotion^(35)^. In the current study, Fe intake was associated with reduced odds of all forms of undernutrition in the older age group. Furthermore, among children, those whose mothers took Fe supplements during pregnancy had lower odds of experiencing wasting or underweight in the younger age group, as well as stunting, wasting or underweight in the older age group. These findings underscore the importance of Fe intake in preventing undernutrition and highlight the potential benefits of Fe supplementation during pregnancy to enhance child health and nutrition outcomes.

Conversely, in the younger age group, taking Fe supplements has been associated with higher odds of experiencing wasting. Similarly, in a study conducted in Panama, supplementing lactating mothers with Fe during the first 6 months of the lactation period was found to be negatively associated with the length-for-age Z score of their children^(36)^. This phenomenon is particularly evident in areas with high parasitic infestations, which contribute to childhood undernutrition. In a double-blind randomised controlled trial conducted in Kenya, 6-month-old children who consumed Fe-fortified maize for 4 months experienced adverse effects on the gut microbiome, increased pathogen abundance and intestinal inflammation due to the increased unabsorbed Fe, which becomes available to the pathogens^(37)^. Food fortification is considered one of the most effective ways to increase nutrient intake in children. However, certain foods, such as maize, contain phytate, an inhibitory compound that reduces the bioavailability of nutrients like Fe^(38)^. In another controlled trial involving Tanzanian preschool children, the intake of high-dose Fe (12·5 mg Fe/d) resulted in increased morbidity and even mortality^(39)^. Furthermore, infants who received Fe-enriched milk for 6 months experienced a significantly higher incidence of diarrhoea^(40)^. However, adding micronutrient powder containing Fe to complementary foods has been shown to reduce Fe deficiency anaemia, even in areas with a high prevalence of infection and inflammation^(41)^. The WHO suggests that in regions with a high prevalence of parasitic infections, combining Fe supplementation with deworming can be an effective approach^(42)^. Research conducted in sub-Saharan Africa revealed that administering Fe supplements and deworming treatments during pregnancy resulted in a decreased likelihood of anaemia and stunted growth among infants under the age of two^(43)^. In the current analysis, deworming was associated with lower odds of wasting or underweight in the older age group.

Our analysis revealed a significant association between using water from an unprotected source and negative effects on stunting, wasting and underweight in both age groups. Additionally, drinking from a bottle on the day prior to data collection was found to be negatively associated with wasting and underweight in the older age group. These findings align with previous studies conducted in Ethiopia and Tanzania^(7,44)^. In regions with inadequate hygiene and sanitation practices, children are at a high risk of contracting parasitic and bacterial infections, which often result in severe diarrhoea. The occurrence of diarrhoea can significantly hinder a child’s growth and development and increase childhood mortality. It has been reported that diarrhoeal disease is responsible for approximately 13·5 % of cases of stunting worldwide. Furthermore, in low- and middle-income countries, the likelihood of stunting increases by 16 % for every 5 % increase in the duration of total episodes of diarrhoea^(45)^. In our analysis, recent episodes of diarrhoea were associated with higher odds of experiencing all forms of undernutrition in both age groups. One of the major causes of hygiene and sanitation-related diarrhoea is the practice of open defecation. Globally, approximately one billion people engage in open defecation, predominantly in low-income countries, particularly in rural areas^(46)^. Mali is one such country where a significant number of people practice open defecation, and children often suffer from recurrent episodes of diarrhoeal diseases, particularly in rural areas^(14)^. The same study reported that in places where access to toilets has improved, child growth has shown significant improvement^(14)^. In the current analysis, residing in rural areas was associated with higher odds of wasting in the younger age group and stunting in the older age group.

The existing guidelines provided by the WHO for managing acute diarrhoea have played a crucial role in significantly reducing mortality rates associated with this condition^(47)^. These guidelines recommend interventions such as rehydration, Zn supplementation, maintaining regular feeding and follow-up care. Zn, in particular, plays a vital role in DNA and RNA metabolism, influencing processes like cell replication, differentiation and growth. Therefore, a deficiency in Zn is associated with various negative health outcomes, including impaired linear growth^(48)^. In a meta-analysis, it was reported that Zn supplementation in infants and early childhood increased height, weight and weight-for-age Z-score^(49)^. In our analysis, the use of Zn supplements for treating diarrhoea was associated with lower odds of stunting and wasting in the younger age group and wasting in the older age group.

Maternal height and BMI demonstrated significant associations with one or more forms of children’s undernutrition. Children born to mothers with shorter stature had higher odds of experiencing stunting in the older age groups. On the other hand, children born to mothers with normal or higher BMI were less likely to experience any form of undernutrition in both age groups. Similar findings have been reported in other African countries^(7,8)^. In low-income countries, maternal BMI can serve as an indicator of food security and overall household well-being. In impoverished environments with limited food availability, the nutritional status of household members, including children, may be similarly affected. Multiple studies have confirmed that maternal BMI is a significant predictor of children’s BMI and has a lasting impact on their nutritional status across generations^(50,51)^. Maternal genetic influence plays a significant role in determining the offspring’s nutritional status, particularly in terms of linear growth, during the critical first 1000 d of life^(51)^. Poor maternal nutrition, inadequate healthcare, and unfavourable environmental conditions can have negative effects on children from an early age and throughout adulthood^(52)^. In our analysis, the current pregnancy status of the mother was negatively associated with stunting or wasting in the younger age group. Early pregnancies often lead to premature weaning, which weakens the immune system and the nutritional status of the children. Moreover, replacing breast milk with less nutrient-rich complementary food exacerbates the nutritional and health issues faced by children^(53)^. In Mali, the primary source of complementary food is derived from plants, with 63 % of the available calories coming from rice, millet, maize and sorghum^(2)^. These cereal crops contain high levels of phytate, which hinder the absorption of vital micronutrients like Zn and Fe, further compromising their nutritional value^(38)^.

In addition to child and maternal factors, socio-economic factors significantly contribute to childhood undernutrition. Research has revealed that children whose mothers have attained at least a secondary education, as well as those belonging to middle or high-income households, are less likely to experience one or more forms of undernutrition. These findings align with similar results reported in other studies^(7,8)^. Hence, it becomes evident that the socio-economic status of families plays a crucial role in determining the nutritional well-being of children. This highlights the importance of addressing economic disparities as a key aspect of effectively combating undernutrition.

### Strengths and limitations

This study contributes to our understanding of child malnutrition in Mali and has the potential to be valuable on a national level for assessing progress in combating child malnutrition. The findings can serve as a crucial resource for planning, implementing, monitoring and evaluating future health promotion programs. However, it is important to acknowledge certain methodological limitations in this study. Since the data were collected from mothers, many of whom had no education, there may be instances of missing, underreported or inaccurately reported important variables related to exposure. Additionally, the cross-sectional nature of the DHS data restricts the ability to establish causal relationships between the explanatory variables and the outcomes presented in the analysis. Finally, we also acknowledge that utilising OR may lead to an overestimation of the effect size. Despite these limitations, the study still provides valuable insights into child undernutrition in Mali and offers a foundation for further research and improvement in addressing this critical issue.

### Conclusion

The progress made in addressing childhood undernutrition in Mali appears to be promising, but it is evident that more comprehensive efforts are needed. Taking a multidisciplinary approach is crucial in addressing the complex factors contributing to undernutrition. This analysis underscores the significant impact of child factors, particularly sanitation and hygiene-related factors, on child outcomes. Moreover, maternal factors play a larger role in the younger age group, whereas household socio-economic factors are more influential in the older age group. This study, like others, demonstrates that children under the age of five are susceptible to similar problems related to undernutrition. However, it is essential to recognise the distinct differences between these two age groups in terms of their nutritional needs, healthcare requirements, parental care and social needs.

Therefore, to successfully alleviate childhood undernutrition in Mali, it is essential to foster collaboration among a diverse range of stakeholders, including governments, non-governmental organisations, communities and international partners. This collaboration should be specifically directed toward education, agriculture, health and nutrition. It is crucial to devise and execute programmes that prioritise the enhancement of children’s health and nutritional status, considering the broader context of their overall well-being. These initiatives should encompass thorough planning, effective implementation, continuous monitoring and rigorous evaluation to ensure their efficacy in addressing the multifaceted challenges of childhood undernutrition in Mali.

## References

[1] Akombi BJ , Agho KE , Merom D et al. (2017) Child malnutrition in sub-Saharan Africa: a meta-analysis of demographic and health surveys (2006–2016). PloS one 12, e0177338. 28494007 10.1371/journal.pone.0177338 PMC5426674

[2] Wuehler SE & Coulibaly M (2011) Situational analysis of infant and young child nutrition policies and programmatic activities in Mali. Matern Child Nutr 7, 83–112. 21410891 10.1111/j.1740-8709.2010.00310.x PMC6860859

[3] FAO, UNICEF, WFP et al. (2020) The State of Food Security and Nutrition in the World 2020. Transforming Food Systems for Affordable Healthy Diets. Rome: FAO.

[4] Leroy JL , Ruel M , Habicht JP et al. (2014) Linear growth deficit continues to accumulate beyond the first 1000 days in low- and middle-income countries: global evidence from 51 national surveys. J Nutr 144, 1460–1466. 24944283 10.3945/jn.114.191981

[5] Black RE , Victora CG , Walker SP et al. (2013) Maternal and child undernutrition and overweight in low-income and middle-income countries. Lancet 382, 427–451. 23746772 10.1016/S0140-6736(13)60937-X

[6] Rytter MJ , Kolte L , Briend A et al. (2014) The immune system in children with malnutrition--a systematic review. PloS one 9, e105017. 25153531 10.1371/journal.pone.0105017 PMC4143239

[7] Gebreegziabher T & Regassa N (2019) Ethiopia’s high childhood undernutrition explained: analysis of the prevalence and key correlates based on recent nationally representative data. Public Health Nutr 22, 2099–2109. 30894232 10.1017/S1368980019000569 PMC10260527

[8] Gewa CA & Yandell N (2012) Undernutrition among Kenyan children: contribution of child, maternal and household factors. Public Health Nutr 15, 1029–1038. 22107729 10.1017/S136898001100245X

[9] Caulfield LE , de Onis M , Blossner M et al. (2004) Undernutrition as an underlying cause of child deaths associated with diarrhea, pneumonia, malaria, and measles. Am J Clin Nutr 80, 193–198. 15213048 10.1093/ajcn/80.1.193

[10] de Wit M , Cairns M , Compaoré YD et al. (2021) Nutritional status in young children prior to the malaria transmission season in Burkina Faso and Mali, and its impact on the incidence of clinical malaria. Malar J 20, 274. 34158054 10.1186/s12936-021-03802-2 PMC8220741

[11] Sundaram ME , Wolfson J , Osterholm M et al. (2020) Meningococcal vaccines and protein-energy undernutrition in children in the African meningitis belt. Vaccine 38, 8351–8356. 33223309 10.1016/j.vaccine.2020.11.012 PMC7751252

[12] Molyneux EM , Walsh AL , Forsyth H et al. (2003) Causes and outcome of bacterial meningitis in Malawian children. Malawi Med J 15, 43–46. 27528955 10.4314/mmj.v15i2.10775 PMC3345437

[13] Makamto Sobgui C , Kamedjie Fezeu L , Diawara F et al. (2018) Predictors of poor nutritional status among children aged 6–24 months in agricultural regions of Mali: a cross-sectional study. BMC Nutr 4, 18. 32153882 10.1186/s40795-018-0225-z PMC7050697

[14] Pickering AJ , Djebbari H , Lopez C et al. (2015) Effect of a community-led sanitation intervention on child diarrhoea and child growth in rural Mali: a cluster-randomised controlled trial. Lancet Glob Health 3, e701–e711. 26475017 10.1016/S2214-109X(15)00144-8

[15] Hatløy A , Hallund J , Diarra MM et al. (2000) Food variety, socioeconomic status and nutritional status in urban and rural areas in Koutiala (Mali). Public Health Nutr 3, 57–65. 10786724 10.1017/s1368980000000628

[16] UNICEF/WHO (2009) UNICEF/WHO Child Growth Standards and the Identification of Severe Acute Malnutrition in Infants and Children. A Joint Statement by the World Health Organization and the United Nations Children’s Fund. Geneva: WHO. 24809116

[17] UNICEF (2020) Conceptual Framework on Maternal and Child Nutrition. Nutrition and Child Development Section. New York: UNICEF.

[18] Rustine O & Rojas G (2006) Guide to DHS Statistics. Demographic and Health Surveys Methodology. P 61. https://dhsprogram.com/pubs/pdf/DHSG1/Guide_to_DHS_Statistics_29Oct2012_DHSG1.pdf (accessed January 2019).

[19] de Onis M , Dewey KG , Borghi E et al. (2013) The World Health Organization’s global target for reducing childhood stunting by 2025: rationale and proposed actions. Matern Child Nutr 9, 6–26. 10.1111/mcn.12075 PMC6860845 24074315

[20] Victora CG , Adair L , Fall C et al. (2008) Maternal and child undernutrition: consequences for adult health and human capital. Lancet 371, 340–357. 18206223 10.1016/S0140-6736(07)61692-4 PMC2258311

[21] Sachdev HS , Fall CH , Osmond C et al. (2005) Anthropometric indicators of body composition in young adults: relation to size at birth and serial measurements of body mass index in childhood in the New Delhi birth cohort. Am J Clin Nutr 82, 456–466. 16087993 10.1093/ajcn.82.2.456

[22] Adair LS (2007) Size at birth and growth trajectories to young adulthood. Am J Hum Biol 19, 327–337. 17421008 10.1002/ajhb.20587

[23] Rahman MS , Howlader T , Masud MS et al. (2016) Association of low-birth weight with malnutrition in children under five years in Bangladesh: do mother’s education, socio-economic status, and birth interval matter? PloS one 11, e0157814. 27355682 10.1371/journal.pone.0157814 PMC4927179

[24] Kusuda S , Fujimura M , Uchiyama A et al. (2012) Trends in morbidity and mortality among very-low-birth-weight infants from 2003 to 2008 in Japan. Pediatr Res 72, 531–538. 22922774 10.1038/pr.2012.114 PMC3547175

[25] Aboagye RG , Ahinkorah BO , Seidu AA et al. (2022) Birth weight and nutritional status of children under five in sub-Saharan Africa. PloS one 17, e0269279. 35679306 10.1371/journal.pone.0269279 PMC9182265

[26] Kai OK & Roberts DJ (2008) The pathophysiology of malarial anaemia: where have all the red cells gone? BMC Med 6, 24. 18717996 10.1186/1741-7015-6-24 PMC2538540

[27] WHO (2011) Hemoglobin Concentrations for the Diagnosis of Anemia and Assessment of Severity. Vitamin and Mineral Nutrition Information Systems. Geneva: World Health Organization (WHO/NMH/NHD/MNM/11.1).

[28] Shifti DM , Chojenta C , Holliday EG et al. (2022) Maternal anemia and baby birth size mediate the association between short birth interval and under-five undernutrition in Ethiopia: a generalized structural equation modeling approach. BMC Pediatr 22, 108. 35227241 10.1186/s12887-022-03169-6 PMC8883659

[29] Ntenda PAM , Nkoka O , Bass P et al. (2018) Maternal anemia is a potential risk factor for anemia in children aged 6–59 months in Southern Africa: a multilevel analysis. BMC Public Health 18, 650. 29788935 10.1186/s12889-018-5568-5 PMC5964691

[30] Bailey RL , West KP Jr & Black RE (2015) The epidemiology of global micronutrient deficiencies. Ann Nutr Metab 66, 22–33. 10.1159/000371618 26045325

[31] Crawley J (2004) Reducing the burden of anemia in infants and young children in malaria-endemic countries of Africa: from evidence to action. Am J Trop Med Hyg 71, 25–34. 15331816

[32] Calis JC , Phiri KS , Faragher EB et al. (2016) Severe anemia in Malawian children. Malawi Med J 28, 99–107. 27895843 PMC5116999

[33] Simbauranga RH , Kamugisha E , Hokororo A et al. (2015) Prevalence and factors associated with severe anaemia amongst under-five children hospitalized at Bugando Medical Centre, Mwanza, Tanzania. BMC Hematol 15, 13. 26464799 10.1186/s12878-015-0033-5 PMC4603816

[34] WHO/UNICEF/UNU (2001) Iron Deficiency Anemia: Assessment, Prevention, and Control. A Guide for Program Managers. Geneva: World Health Organization WHO/NHD/013.

[35] Hentze MW , Muckenthaler MU , Galy B et al. (2010) Two to tango: regulation of Mammalian iron metabolism. Cell 142, 24–38. 20603012 10.1016/j.cell.2010.06.028

[36] González-Fernández D , Nemeth E , Pons EDC et al. (2022) Multiple indicators of undernutrition, infection, and inflammation in lactating women are associated with maternal iron status and infant anthropometry in Panama: the MINDI Cohort. Nutrients 14, 3497. 36079755 10.3390/nu14173497 PMC9460351

[37] Jaeggi T , Kortman GA , Moretti D et al. (2015) Iron fortification adversely affects the gut microbiome, increases pathogen abundance and induces intestinal inflammation in Kenyan infants. Gut 64, 731–742. 25143342 10.1136/gutjnl-2014-307720

[38] Rahman S & Shaheen N (2022) Phytate-iron molar ratio and bioavailability of iron in Bangladesh. Trop Med Int Health 27, 509–514. 35383403 10.1111/tmi.13750 PMC9322336

[39] Sazawal S , Black RE , Ramsan M et al. (2006) Effects of routine prophylactic supplementation with iron and folic acid on admission to hospital and mortality in preschool children in a high malaria transmission setting: community-based, randomised, placebo-controlled trial. Lancet 367, 133–143. 16413877 10.1016/S0140-6736(06)67962-2

[40] Power HM , Heese HD , Beatty DW et al. (1991) Iron fortification of infant milk formula: the effect on iron status and immune function. Ann Trop Paediatr 11, 57–66. 1714697 10.1080/02724936.1991.11747479

[41] Macharia-Mutie CW , Moretti D , Van den Briel N et al. (2012) Maize porridge enriched with a micronutrient powder containing low-dose iron as NaFeEDTA but not amaranth grain flour reduces anemia and iron deficiency in Kenyan preschool children. J Nutr 142, 1756–1763. 22810982 10.3945/jn.112.157578

[42] WHO (2021) Deworming Women during Pregnancy has a Positive Effect on Child Survival and Health. Geneva: World Health Organization (WHO).

[43] Traore SS , Bo Y , Kou G et al. (2023) Iron supplementation and deworming during pregnancy reduces the risk of anemia and stunting in infants less than 2 years of age: a study from Sub-Saharan Africa. BMC Pregnancy Childbirth 23, 63. 36698082 10.1186/s12884-023-05399-7 PMC9875517

[44] Chirande L , Charwe D , Mbwana H et al. (2015) Determinants of stunting and severe stunting among under-fives in Tanzania: evidence from the 2010 cross-sectional household survey. BMC Pediatr 15, 165. 26489405 10.1186/s12887-015-0482-9 PMC4618754

[45] Danaei G , Andrews KG , Sudfeld CR et al. (2016) Risk factors for childhood stunting in 137 developing countries: a comparative risk assessment analysis at global, regional, and country levels. PLoS Med 13, e1002164. 27802277 10.1371/journal.pmed.1002164 PMC5089547

[46] WHO (2016) Water Sanitation Hygiene. Geneva: WHO.

[47] WHO/UNICEF (2023) Zinc Supplementation in the Management of Diarrhea. https://www.who.int/tools/elena/interventions/zinc-diarrhoea (accessed August 2023).

[48] Imdad A & Bhutta ZA (2011) Effect of preventive zinc supplementation on linear growth in children under 5 years of age in developing countries: a meta-analysis of studies for input to the lives saved tool. BMC Public Health 11, S22. 21501440 10.1186/1471-2458-11-S3-S22 PMC3231896

[49] Lourenco BH , Villamor E , Augusto RA et al. (2015) Influence of early life factors on body mass index trajectory during childhood: a population-based longitudinal analysis in the Western Brazilian Amazon. Matern Child Nutr 11, 240–252. 23020806 10.1111/mcn.12005 PMC6860355

[50] Deierlein AL , Siega-Riz AM , Adair LS et al. (2011) Effects of pre-pregnancy body mass index and gestational weight gain on infant anthropometric outcomes. J Pediatr 158, 221–226. 20863516 10.1016/j.jpeds.2010.08.008 PMC3017634

[51] Young MF & Nguyen PH (2018) Role of maternal preconception nutrition on offspring growth and risk of stunting across the first 1000 days in Vietnam: a prospective cohort study. PloS one 13, e0203201. 30161206 10.1371/journal.pone.0203201 PMC6117029

[52] Schack-Nielsen L , Michaelsen KF , Gamborg M et al. (2010) Gestational weight gain in relation to offspring body mass index and obesity from infancy through adulthood. Int J Obes 34, 67–74. 10.1038/ijo.2009.206 19918246

[53] Oddy WH (2017) Breastfeeding, childhood asthma, and allergic disease. Ann Nutr Metab 70, 26–36. 10.1159/000457920 28521318

